# The Role of Tβ4-POP-Ac-SDKP Axis in Organ Fibrosis

**DOI:** 10.3390/ijms232113282

**Published:** 2022-10-31

**Authors:** Wei Wang, Wenning Jia, Chunping Zhang

**Affiliations:** 1 Queen Mary School, Nanchang University, Nanchang 330006, China; 2 Department of Cell Biology, College of Medicine, Nanchang University, Nanchang 330006, China

**Keywords:** Tβ4, POP, Ac-SDKP, fibrosis

## Abstract

Fibrosis is a pathological process in which parenchymal cells are necrotic and excess extracellular matrix (ECM) is accumulated due to dysregulation of tissue injury repair. Thymosin β4 (Tβ4) is a 43 amino acid multifunctional polypeptide that is involved in wound healing. Prolyl oligopeptidase (POP) is the main enzyme that hydrolyzes Tβ4 to produce its derivative N-acetyl-seryl-aspartyl-lysyl-proline (Ac-SDKP) which is found to play a role in the regulation of fibrosis. Accumulating evidence suggests that the Tβ4-POP-Ac-SDKP axis widely exists in various tissues and organs including the liver, kidney, heart, and lung, and participates in the process of fibrogenesis. Herein, we aim to elucidate the role of Tβ4-POP-Ac-SDKP axis in hepatic fibrosis, renal fibrosis, cardiac fibrosis, and pulmonary fibrosis, as well as the underlying mechanisms. Based on this, we attempted to provide novel therapeutic strategies for the regulation of tissue damage repair and anti-fibrosis therapy. The Tβ4-POP-Ac-SDKP axis exerts protective effects against organ fibrosis. It is promising that appropriate dosing regimens that rely on this axis could serve as a new therapeutic strategy for alleviating organ fibrosis in the early and late stages.

## 1. Tβ4-POP-Ac-SDKP Axis

Thymosin β4 (Tβ4) is a water-soluble peptide with a highly conserved structure composed of 43 amino acid residues and was first found in the calf thymus extract [1]. It is also a multifunctional peptide that can stimulate angiogenesis, promote cell proliferation, inhibit apoptosis, reduce inflammation, and inhibit scar formation and fibrosis [2]. Tβ4 is crucial in tissue repair and regeneration, and it binds to G-actin and inhibits its polymerization to promote cell migration, including stem/progenitor cell mobilization, migration, and differentiation to form new blood vessels and regenerate tissue [3,4]. After the injury, Tβ4 is released by many types of cells such as platelets and macrophages and exerts anti-inflammatory effects by reducing the number of inflammatory cells and downregulating the expression levels of many inflammatory factors such as tumor necrosis factor-α (TNF-α), interleukin (IL)-1β, and IL-6, as well as inhibiting nuclear factor-κB (NF-κB) expression [5]. Studies have found that Tβ4 is involved in the repair and treatment of skin wounds, dry eyes, myocardial infarction (MI), brain injury, and other injuries in various tissues and organs [6]. It reduces the number of myofibroblasts in wounds, thereby inhibiting scar formation and fibrosis [7]. Tβ4 has an anti-fibrotic effect and can treat fibrosis of the liver, lung, and kidney [2].

**Figure 1:**
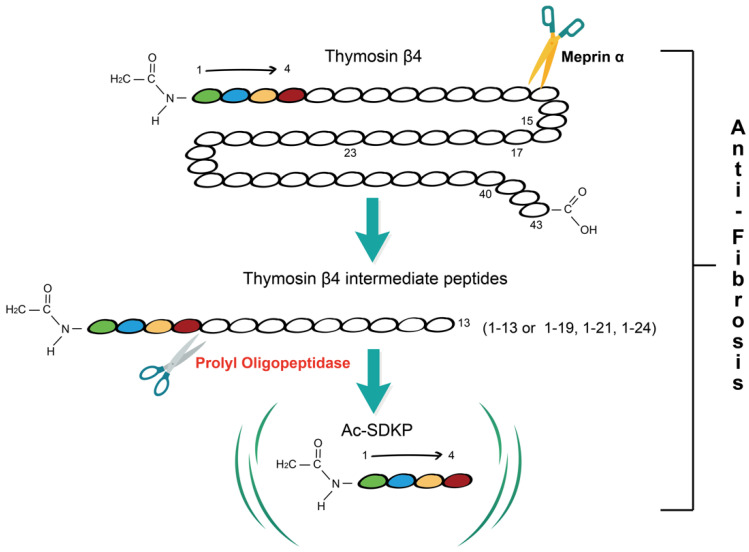
Overview of Tβ4-POP-Ac-SDKP axis. Thymosin β4 (Tβ4), a 43-amino-acid peptide, is firstly cleaved by meprin α into Tβ4 intermediate peptides shorter than 30 amino acids in length and these peptides are hydrolyzed by prolyl oligopeptidase (POP) to produce the N-terminal tetrapeptide N-acetyl-seryl-aspartyl-lysyl-proline (Ac-SDKP). Tβ4 intermediate peptides 1-13, 1-19, 1-21, and 1-24 are products of Tβ4 hydrolysis at four specific meprin α cleavage sites. The Tβ4-POP-Ac-SDKP axis has an anti-fibrotic effect.

Prolyl oligopeptidase (POP) is a serine protease that can specifically hydrolyze peptide bonds at the carboxyl-terminal of proline residues in polypeptide chains [8]. After the cleavage of Tβ4 into NH_2_-terminal intermediate peptides less than 30 amino acids in length by metalloprotease meprin α, POP hydrolyzes these intermediate peptides to ultimately release N-acetyl-seryl-aspartyl-lysyl-proline (Ac-SDKP) [9] (Figure 1). Studies have shown that POP is widely distributed in mammalian organs and has an anti-fibrotic effect. It generates Ac-SDKP, which has been demonstrated to alleviate liver fibrosis in carbon tetrachloride (CCl_4_)-induced fibrosis in a rat model and attenuate the activation of primary hepatic stellate cells (HSCs) isolated from rats in vitro [10,11]. In the kidney and heart, POP inhibitor S17092 treatment decreased the endogenous Ac-SDKP and increased collagen deposition in vivo [12], suggesting that POP is involved in alleviating fibrosis through Tβ4-POP-Ac-SDKP axis.

Ac-SDKP, an N-terminal derivative of Tβ4, is a tetrapeptide generated by the hydrolysis of its precursor Tβ4 by meprin α and POP [9]. In vivo, Ac-SDKP is degraded by the N-terminal catalytic sites of angiotensin-converting enzyme (ACE) to become inactive, and its plasma concentration is increased five times when given ACE inhibitors [13]. Initial studies believed that Ac-SDKP prevents hematopoietic stem cells from entering the S phase spontaneously, keeping them in the G0/G1 phase and results in the inhibition of hematopoietic stem cell proliferation [14,15]. More and more research in recent years has discovered that it can inhibit fibroblast proliferation and fibrosis in various organs and tissues such as the liver, kidney, heart, and lung. In the liver, heart, and lung, Ac-SDKP reduces collagen synthesis by downregulating the transforming growth factor (TGF)-β1 and reducing the differentiation of fibroblasts into active myofibroblasts [16,17,18]. In the kidney, Ac-SDKP attenuates renal dysfunction and fibrosis after anti-glomerular basement membrane nephritis is developed, which is associated with inhibiting the infiltration of macrophages and the TGF-β/Smad pathway [19].

The Tβ4-POP-Ac-SDKP axis is widely present in various tissues and organs including the liver, kidney, heart, and lung, and it is crucial for the regulation of tissue and organ fibrosis.

## 2. Tissue and Organ Fibrosis

Tissue and organ fibrosis refers to a pathological process in which connective tissue components are excessively accumulated and it is the result of the dysregulated repair response after tissue injury [20]. Fibrosis is a significant factor in the occurrence and progression of diseases in major organs of the human body, such as the liver, kidney, heart, and lung [21]. Continued progression of fibrosis can lead to organ structural damage, loss of function, and ultimately failure. Tissue and organ fibrosis are the main causes of many diseases and deaths [22]. The essence of fibrosis is the repair response of tissues after injury to protect the relative integrity of tissues and organs. Tissue injury can lead to tissue cell degeneration, necrosis, and inflammatory response. If the damage is small, normal parenchymal cells around the damaged cells will undergo proliferation and repair, which can completely restore normal structure and function [23]. However, if the damage is serious or the repeated damage exceeds the regeneration capacity of the parenchymal cells around the injury site, the extracellular matrix (ECM) will proliferate to repair the defect tissue, that is, the pathological changes of fibrosis will occur [24]. Excessive deposition of collagen and other extracellular matrix proteins repair the defect but do not have the structure and function of the original organ parenchymal cells. It can result in fibrosis and decreased organ function if the healing reaction is excessive and out of control [25].

Fibrogenesis is a highly organized process that is regulated by various chemical signals and cells. Parenchymal cells are damaged following injury while immune cells dominated by macrophages are activated. Large amounts of biological mediators such as IL-4, IL-13, IL-25, IL-33, platelet-derived growth factor (PDGF), and other cytokines and chemokines secreted by these immune cells cause mesenchymal cells to become active locally which transform fibroblasts, vascular smooth muscle cells, pericytes, mesothelial cells, fibrocytes and many other types of cells into myofibroblasts, which express α-smooth muscle actin (α-SMA) [26,27]. Myofibroblasts involve in extracellular matrix production leading to scar formation and the destruction of tissue and organ structure [28]. Moreover, recent studies showed that scleroderma-associated fibroblasts (ScAFs) expressing the LGR5 receptor and circulating fibrocytes are associated with skin fibrosis progression [29,30]. In addition to producing ECM proteins, myofibroblasts aid in repair by producing contractile forces that are conveyed to the surrounding ECM and activate TGF-β, a crucial cytokine in fibrosis [31]. Furthermore, it has been demonstrated that the activity of fibroblast and the process of fibrosis are regulated by the synergistic or antagonistic action of other various cytokines such as C-C motif chemokine 2 (CCL2), monocyte chemoattractant protein-1 (MCP-1), interleukins, TNF-α, reactive oxygen species (ROS) [32].

Many studies have reported that Tβ4-POP-Ac-SDKP axis plays an important role in the development of tissue and organ fibrosis. The research on the regulatory mechanism of Tβ4-POP-Ac-SDKP is conducive to further understanding the relationship between tissue damage repair imbalance and the occurrence and development of tissue and organ fibrosis, as well as underlying pathophysiological mechanisms. We will summarize the effect of Tβ4-POP-Ac-SDKP axis on fibrosis of different organs including the liver, kidney, lung, heart, and the underlying mechanisms of fibrogenesis. 

## 3. Liver

Liver fibrosis is a chronic liver injury process. During the injury, the activation of HSCs contributes to the ECM deposition [33]. The Tβ4-POP-Ac-SDKP axis regulates the pathogenesis of liver fibrosis by participating in the activation of HSCs. CCl_4_ acts as the hepatotoxic agent used to create liver injury and fibrosis mouse models such as hepatotoxicity in humans which is marked by hepatic lobule necrosis [11]. Bile duct ligation (BDL) is a method that induces cholestasis and creates a model of liver fibrosis [34].

Several studies have shown that endogenous Tβ4 is upregulated in the damaged liver and might promote the process of fibrosis. Li et al. reported Tβ4 increased in CCl_4_-induced acute injury mice and BDL-induced mice [35]. The activity of POP which converted Tβ4 into Ac-SDKP is higher in the normal liver than in the chronically injured liver. According to Kim et al., decreased POP activity in damaged livers of CCl_4_-treated mice at 6 and 10 weeks induced upregulation of endogenous Tβ4 and the accumulation of Tβ4 protein [36]. Further study showed that in LX-2 cells, the activated human HSC line, the HSC functions were inhibited after Tβ4 siRNA treatment, and the knockout of Tβ4 blocked the transdifferentiation of HSCs. Tβ4 activated the smoothened (SMO) and glioblastoma 2 (GLI2) pathway to regulate hedgehog (Hh) signaling and promoted fibrosis by activating HSCs in CCl_4_-treated Tβ4-overexpressing transgenic mice [37]. However, there are studies showing endogenous Tβ4 exerted an anti-fibrotic effect on liver fibrosis. Li et al. reported that the immunohistochemistry (IHC) results showed the expression of Tβ4 in human cirrhotic liver tissues was lower compared with normal tissues. Pre-treatment of adeno-associated virus-Tβ4 (AAV-Tβ4) ameliorated fibrosis, HSC activation, and the expressions of pro-fibrotic cytokines such as TGF-β1, plasminogen activator inhibitor-1 (PAI-1), connective tissue growth factor (CTGF), PDGF-B in CCl_4_-induced mice [35].

Many studies have suggested that exogenous Tβ4 exerts a protective role in liver injury and fibrosis. Gordillo et al. found that Tβ4 relieved liver damage in an acute injury rat model after CCl_4_ treatment 24h shown by hematoxylin-eosin (H&E) staining through suppressing inflammatory infiltration, necrosis, and increasing the mRNA expression of α-SMA, α1(and 2) collagen, PDGF-β receptor (PDGF-βr), and fibronectin. In this model, IHC images showed that Tβ4 attenuated HSC activation by restoring the expression of peroxisome proliferator-activated receptor γ (PPARγ) which is a quiescent sign of HSCs, and downregulating methyl-CpG binding protein 2 (MECP2) mRNA expression, which was significantly expressed in activated HSCs [38]. Li et al. reported that exogenous Tβ4 attenuated acute liver damage in CCl_4_-treated mice and chronic fibrosis in CCl_4_-treated rats as demonstrated by H&E staining. The Tβ4+CCl_4_ group showed low expression of NF-κB p65, an indicator of inflammation, and suppression of oxidative stress compared with the CCl_4_ group, which suggested the protective function of exogenous Tβ4 on damaged liver [39]. Yang et al. demonstrated that exogenous Tβ4 abolished the phosphorylation of NF-κB p65 induced by lncRNA-p21 and reversed the fibrosis in Ad-p21 mice by inhibiting PI3K-AKT-NF-κB pathway [40]. Zhu et al. also reported that Tβ4 significantly inhibited IL-1β-induced HSC-LX2 cell proliferation by preventing the activation of the NF-κB pathway and downregulating the expression of p-IKB and translocation of NF-κB p65 [41]. Further studies demonstrated that Tβ4-depleted LX-2 cells upregulated circular RNA (circRNA)-0067835, a sponge of microRNA (miR)-155, and increased AKT/FOXO3a signaling, which promoted liver fibrosis [42]. In vitro, Karina et al. demonstrated that Tβ4 blunted the binding of AKT to actin and subsequently inhibited AKT phosphorylation which blocked the expression of PDGF-βr (a marker of HSC activation), preventing the activation and migration of cultured human HSCs [43]. Barnaeva et al. reported that Tβ4 prevented the proliferation of cultured human HSCs in vitro by upregulating the expression of hepatocyte growth factor (HGF), which could ameliorate fibrosis, and downregulating the expression of PDGF-βr [44]. Tβ4 was also identified to bind to PDGF-BB directly and block its binding to PDGF-βr, which prevented human HSC activation in vitro [45]. Chen et al. demonstrated that Tβ4 alleviated liver fibrosis in BDL-induced mice by downregulating the TGF-β1/Smad pathway [46]. However, Hong et al. suggested that Tβ4 alleviated CCl_4_-induced liver fibrosis of mice by downregulating the Notch signaling, Notch2, and Notch3, rather than regulating TGF-β signaling pathway [47]. Li et al. reported that Tβ4 reduced the activation of TGF-β1-induced HSCs and the expression of profibrogenic factors such as TGF-β1, PDGF-B, CTGF, and PAI-1 in LX-2 cells (human HSC cell line), HSC-T6 cells (rat hepatic stellate cell line), and LO2 cells (human embryo liver cell line). Exogenous Tβ4 also reduced the expression of TGF-β receptor-II (TGF-βRII) in fibrotic mice liver tissues, cultured LX-2, and LO2 cells. Hepatocytes and HSCs (the major source of fibrogenic myofibroblasts) together participate in liver fibrosis. When the liver is damaged, inflammatory and necrotic hepatocytes will release some cytokines such as TGF-β1 and TNF-α, which promote HSC activation, producing ECM, and causing liver fibrosis [48]. The expression of TGF-βRII was upregulated when endogenous Tβ4 was neutralized by anti-Tβ4 antibody in LX-2 and LO2 cells [35]. 

POP may have anti-fibrotic effects mediated by Ac-SDKP. CCl_4_-treated rats caused the expression of Tβ4 downregulated in the early phase and the activity of POP decreased in a time-dependent manner, inducing a reduced release of endogenous Ac-SDKP. The defect of Tβ4-POP-Ac-SDKP axis caused liver fibrosis, which could be relieved by exogenous Ac-SDKP. It was demonstrated that the activation of primary HSCs of rats was inhibited by exogenous Ac-SDKP [10]. Zhou et al. reported that the concentration of intracellular Ac-SDKP decreased with decreasing POP activity caused by S17092 administration and increased after POP-expressing lentivirus transduction in HSC-T6 cells. POP and Ac-SDKP exerted an inhibitory effect on the activation or proliferation of HSCs respectively with different effects on TGF-β signaling. POP inhibited the expression of TGF-β1 by upregulating PPARγ and Smad7 in HSC-T6 cells whereas Ac-SDKP suppressed the TGF-β1 signaling by downregulating the TGF-β1 and p-Smad2/3 [49]. Zhang et al. also demonstrated that exogenous Ac-SDKP mitigated BDL-induced liver fibrosis by downregulation of TGF-β1 and upregulation of bone morphogenetic protein-7 (BMP-7) which counteracted TGF-β1 [16]. Wei et al. reported that Ac-SDKP was downregulated in a CCl_4_-treated liver fibrosis rat model, and exogenous Ac-SDKP caused the decreased expressions of Wilms’ tumor 1-associated protein (WTAP) and N^6^-methyladenosine (m^6^A), which suppressed the Hh signaling, alleviated the HSCs apoptosis and fibrosis [50].

## 4. Kidney

Kidney fibrosis is a tissue repair process in progressive kidney diseases, including glomeruli and interstitial fibrosis. The major causes of fibrosis are chronic kidney diseases such as diabetic nephropathy, hypertension, and lupus nephritis [51]. In the process of renal fibrogenesis, fibroblasts or myofibroblasts are activated by profibrotic cytokines and secrete ECM. Tubular cells and endothelial cells develop a profibrotic phenotype via epithelial–mesenchymal transition (EMT) or endothelial–mesenchymal transition (EndMT) pathway and are responsible for the production of ECM [52].

The Tβ4-POP-Ac-SDKP axis has a significant impact on renal fibrosis. Endogenous Tβ4 improves the pathogenesis of kidney interstitial fibrosis. Tβ4 mainly exists in podocytes of mouse glomeruli. The nephrotoxic serum (NTS)-induced nephritis mouse model is a nephrotoxic model to induce crescentic glomerulonephritis leading to fibrosis [53]. Vasilopoulou et al. built an NTS-induced nephritis model with Tmsb4x^-/-^ mice. The lack of Tβ4 gene (Tmsb4x) aggravated the damage of glomeruli, causing the podocyte redistribution to the Bowman capsule and worsening the inflammation around glomeruli and interstitial fibrosis [54]. Ac-SDKP also exerted an antiproliferative effect by controlling the cell cycle in two renal cell lines, renal proximal tubular epithelial cells (LLC-PK1), and renal interstitial fibroblasts cell line (NRK 49F) which were important in renal interstitial fibrosis [55].

POP inhibitor prevented the transformation from Tβ4 to Ac-SDKP; furthermore, Romero et al. suggested that the chronic infusion of POP inhibitor KYP-2047 to rat model decreased the concentration of Ac-SDKP mainly in the distal nephron and worsened the kidney medullary fibrosis, which was rescued by infusion of KYP-2047 and Ac-SDKP simultaneously [56]. In addition, in a rat model administrated by S17092, decreased endogenous levels of Ac-SDKP aggravated excessive collagen deposition and glomerulosclerosis [12].

Unilateral ureteric obstruction (UUO) is a method to develop end-stage renal disease leading to tubulointerstitial fibrosis. In UUO C57Bl/6 and PAI-1 knockout C57Bl/6 model, endogenous Tβ4 was significantly upregulated and accompanied with interstitial fibrosis. Tβ4 treatment decreased late-stage fibrosis, but Tβ4+POP inhibitor exerted a profibrotic effect. Both effects disappeared in PAI-1 knockout mice, which suggests the important role of PAI-1 on Tβ4. However, Ac-SDKP treatment improved the early- and late-stage fibrosis in both wild-type and PAI-1 knockout mice [57]. Furthermore, Xu et al. showed that intrinsic Tβ4 was upregulated in early-stage glomerulosclerosis induced by 5/6 nephrectomy, and this was related to the expression of a profibrogenic factor, angiotensin II (Ang II)-induced PAI-1 in cultured glomerular endothelial cells. PAI-1 can prevent the plasminogen activation and cause matrix deposition and fibrosis [58]. In UUO BALB/c mice, Ac-SDKP and ACE inhibitor captopril treatment downregulated expressions of collagen IV, α-SMA, and MCP-1, therefore reducing the fibrosis [59]. Thus, the Tβ4-POP-Ac-SDKP axis plays an important role in renal fibrosis in the UUO model. Previous studies have shown that the molecular mechanism of this axis in tubulointerstitial fibrosis. TGF-β is a central factor involved in fibrosis. Yuan et al. reported that in UUO rats, Tβ4 had a dose-dependent protective effect on renal fibrosis and apoptosis of NRK-52E, a cell line of tubular epithelial cells, via downregulation of TGF-β pathway [60]. Kanasaki et al. showed that in human mesangial cells, Ac-SDKP inhibited the TGF-β-induced expression of PAI-1, Smad2 phosphorylation, and promoted the translocation of Smad7 from the cytoplasm to nucleus, which inhibited R-Smad proteins [61]. Wang et al. reported that in UUO rats, Ac-SDKP treatment relieved interstitial fibrosis through the downregulation of TGF-β and α-SMA [62]. Chan et al. reported that in UUO BALB/c mice, the level of AC-SDKP in urine was elevated by captopril treatment and reduced with co-treatment of captopril and S17092. The increased endogenous Ac-SDKP significantly ameliorated the interstitial fibrosis by inhibiting TGF-β pathway and p44/42 mitogen-activated protein kinase (MAPK) pathway [63].

Diabetic nephropathy is a common progressive kidney disease developed to kidney fibrosis. The Tβ4 levels and the expression of POP were significantly decreased in streptozotocin (STZ)-treated CD-1 mice with severe kidney liver fibrosis [52]. The S17092 significantly inhibited the Ac-SDKP synthesis, resulting in the central metabolism disruption and kidney fibrosis. In S17092-injected STZ-induced diabetic C57Bl6 mice, Ac-SDKP treatment improved the metabolism of myofibroblasts and reduced the accumulation of collagen and fibronectin compared with the no treatment group [64]. In STZ-induced diabetic nephropathy rats, exogeneous Ac-SDKP reduced renal fibrosis, and addition of ACE inhibitor ramipril further improved the fibrosis by repressing the metabolism of Ac-SDKP [65]. Some studies have demonstrated that EMT or EndMT plays an important role in the fibrosis of diabetic models. Ac-SDKP may suppress fibrogenesis via inhibition of EMT or EndMT. Fibroblast growth factor (FGF), a ligand binding to FGF receptor (FGFR), plays an important role in the survival and EndMT of endothelial cells [66]. Li et al. demonstrated that Ac-SDKP had anti-EndMT effects dependent on FGFR1 signaling. In STZ-induced diabetic endothelial-specific FGFR1 knockout mice (FGFR1^EKO^), the knockout of FGFR1 had resistance to the anti-fibrotic effect of Ac-SDKP and developed severe fibrosis compared with the control group. Ac-SDKP did not affect FGFR-dependent EndMT in the kidney but had a partial suppressive effect on EMT. It prevented the TGF-β-dependent EMT which was stimulated by endothelial FGFR1-deficiency induced EndMT in the human proximal tubule epithelial cell line [67]. Gao et al. also reported that FGFR1 expressed on the cell membrane exerted an anti-EndMT function of Ac-SDKP via forming FGFR1–βklotho (KLB) complex with its co-receptor KLB in cultured vascular endothelial cells. FGF19 or FGF21 was the ligand of the FGFR1–KLB complex and prevented EndMT through downregulation of the MEK/ERK pathway [68]. Otherwise, Ac-SDKP can regulate EndMT and anti-fibrotic program via crosstalk with microRNA network. MiR-let-7 and miR-let-29 were induced by Ac-SDKP [69]. A study showed that in cultured endothelial cells, human dermal microvascular endothelial cells (HMVECs), upregulated miR-let-7 inhibited the TGF-β pathway and increased expression of miR-let-29. Meanwhile, increased miR-let-29 suppressed interferon-γ (IFN-γ), which activated the FGF pathway and in turn induced miR-let-7 [70]. Nagai et al. also demonstrated that in STZ-induced CD-1 diabetic mice and cytokines-induced cultured endothelial cells, a model of EndMT, Ac-SDKP+ACE inhibitor imidapril decreased fibrosis and EndMT by restoring the level of FGF-induced miR-let-7, which was suppressed in renal fibrosis [71]. Srivastava et al. reported that Ac-SDKP treatment increased the stimulative effects of imidapril on the miR-let-29 and miR-let-7 in cultured HMVECs, which inhibited DPP-4, a key molecule related to kidney fibrosis, and the TGF-β pathway [72]. 

Hypertension can result in renal impairment with the characteristics of inflammation, fibrosis, and proteinuria, and hypertensive renal injury (HRI) is a main cause of end-stage renal diseases [73,74,75,76,77]. Studies have demonstrated that Ac-SDKP exerts a protective effect against HRI. Liao et al. reported that Ac-SDKP treatment greatly prevented and reversed albuminuria and renal fibrosis as well as improved renal function in 5/6 Nephrectomy (5/6Nx)-induced hypertensive rat model and these events were associated with a decrease in inflammation, glomerulosclerosis, and an increase in the glomerular slit pore protein, nephrin [78]. Rhaleb et al. found that in C57BL/6J mice with deoxycorticosterone acetate (DOCA)-salt-induced hypertension, Ac-SDKP inhibited renal collagen content, macrophage infiltration, nephrin expression, and albuminuria [79].

## 5. Heart

Cardiac fibrosis is characterized by excessive deposition of myocardial ECM mainly composed of collagen [80]. It is a pathological change secondary to various acute injuries and chronic diseases and is common in the late stage of various heart diseases such as MI and hypertension [81]. Various cell types are involved in the cardiac fibrosis process, in which ECM is mainly produced by cardiac fibroblasts [82].

Tβ4 has a cardioprotective effect following injury. Kumar et al. found that the amount of collagen deposited in the heart of Tβ4 knockout mice and wild type mice did not differ significantly. However, in Ang II-induced cardiac damage C57BL/6 mice, Tβ4 knockout led to an increase in profibrotic α-SMA expression and fibrosis [83]. Histochemical staining showed that treatment with Tβ4 suppressed collagen synthesis in the heart of C57BL/6 mice with MI [84]. Evans et al. reported that Tβ4 reduced fibrosis of the left ventricular wall in post MI mice shown by magnetic resonance imaging [85]. In cardiac fibroblasts treated with H_2_O_2_ in vitro, Tβ4 inhibited the mRNA expression levels of profibrotic genes such as CTGF, collagen I, and III [86]. The transplantation of embryonic stem cells overexpressing Tβ4 to the MI mice improved the generation of cardiac fibrosis by inhibiting MMP-9 activation [87]. Sopko et al. also discovered that Tβ4 reduced collagen I and III expressions and NF-kB activation while upregulating the PINCH-1-ILK-α-parvin (PIP) complex and Akt activation in MI mice [88]. However, Stark et al. found that intraperitoneal injection exogenous Tβ4 did not reduce myocardial fibrosis of the left ventricle significantly in pegylated and liposomal formulations of doxorubicin (PLD)-induced cardiotoxic FVB/n mice model [89].

Chronic administration of an oral S17092 that prevented the release of Ac-SDKP from Tβ4 significantly reduced cardiac endogenous levels of Ac-SDKP in normal rats and induced perivascular fibrosis and collagen deposition, indicating that the Tβ4-POP-Ac-SDKP axis regulates the amount of collagen in the heart [12].

Ac-SDKP reduces collagen deposition in rats with hypertension, MI, or radiation induction in vivo and prevents cardiac fibroblasts from producing collagen in vitro.

A study indicated that the collagen content and collagen volume fraction of the left ventricle (LV) were decreased with the treatment of Ac-SDKP both in spontaneously hypertensive rats and normal rats [90]. Rhaleb et al. reported that Ac-SDKP inhibited the proliferation of cultured primary cardiac fibroblasts isolated from rats and collagen synthesis in aldosterone and salt-induced hypertension rat model with the characteristic of fibrosis [91]. Moreover, in rats with heart failure after MI, exogeneous Ac-SDKP administrated before induction of MI or after MI decreased total collagen content, cardiac interstitial collagen fraction (ICF), and perivascular collagen deposition, suggesting that Ac-SDKP can prevent the development of myocardial fibrosis as well as reverse the established fibrosis [92]. Sharma et al. reported that chronic Ac-SDKP treatment ameliorated coronary vascular fibrosis and exerted a cardioprotective effect by decreasing macrophage activation, inflammation, and fibrosis in a rat model of ionizing radiation-induced cardiotoxicity [93,94]. 

Many studies demonstrated that the antifibrotic effect of Ac-SDKP in the heart was mediated by interference with the TGF-β signaling pathway both in vivo and in vitro. TGF-β/Smad signaling pathway plays an important role in fibroblast proliferation and ECM accumulation and is implicated in many fibrotic diseases [95]. Yang et al. demonstrated that Ac-SDKP downregulated TGF-β in rats after MI and thus inhibiting the differentiation of cardiac fibroblasts to myofibroblasts, which is important in ECM production [92]. A study carried out by Peng et al. found that Ac-SDKP dramatically reduced the enhanced LV fibrosis in 2-kidney, 1-clip (2K-1C) hypertensive rats and was caused by reduction of TGF-β and its mediator CTGF production [96]. Rasoul et al. found that the antifibrotic effect of Ac-SDKP on the LV of Ang II-induced hypertensive rat model was due to decreasing the expression of TGF-β and CTGF [97]. Decreased collagen cross-linking and total collagen when treated with Ac-SDKP was also caused by a decrease in TGF-β1, LOXL1, and the infiltration of lymphocytes and macrophages in rats of Ang II-induced hypertension [98]. Ac-SDKP reduced aortic fibrosis in Ang II-induced hypertensive rats associated with inhibition of protein kinase C activation resulting in a reduction in oxidative stress, inflammation, TGF-β1 expression, and Smad2 phosphorylation [99]. In diabetic rats, Castoldi et al. reported that Ac-SDKP decreased interstitial and perivascular fibrosis in the left ventricle, and these effects were associated with a decreased level of TGF-β1 and phosphorylated Smad2 and Smad3 [100]. In addition, in L1173 and L1172 transgenic rat lines overexpressing ACE in the myocardium, decreased endogenous Ac-SDKP enhanced myocardial collagen synthesis by promoting the phosphorylation of Smad2 and Smad3 in the ACE gene-dose-dependent manner [101]. Treatment with Ac-SDKP inhibited myocardial fibrosis in intrapericardial galectin-3-induced myocarditis rats through decreasing TGF-β expression and Smad3 phosphorylation [102]. In vitro, research indicated that Ac-SDKP also exerted anti-fibrotic effects by inhibiting the phosphorylation and nuclear translocation of Smad2 in cardiac fibroblasts [103]. 

Additionally, Ac-SDKP is involved in regulating other pathways to exert an anti-fibrotic effect in the heart. In the ALDO-salt-induced hypertensive rat model, Ac-SDKP administration decreased LV interstitial and perivascular collagen deposition which are mediated by blocking p42/44 MAPK phosphorylation [104]. Rhaleb et al. demonstrated that Ac-SDKP inhibited DNA synthesis and ET-1-induced collagen generation in primary cardiac fibroblasts derived from adult rats associated with reducing the activity of the p42/44 MAPK pathway [91]. They also found that Ac-SDKP alleviated the increased expression level of MMP-2, MMP-9, and MMP-13 which was induced by IL-1β in adult rat cardiac fibroblasts in vitro resulting from interfering with p42/44 MAPK pathway and NF-κB activation [105]. In human coronary artery endothelial cells, Ac-SDKP pretreatment decreased the expression of TNF-α-induced leukocyte adhesion molecule-1 (ICAM-1) in a dose-dependent manner by inhibiting NF-κB pathway, which prevents inflammation and fibrosis [106].

## 6. Lung

Pulmonary fibrosis is a progressive, irreversible pulmonary interstitial disease characterized by progressive dyspnea and even respiratory failure [107,108]. It is classified into more than 200 types, of which idiopathic pulmonary fibrosis (IPF) of unknown causes is the most common form [109,110]. Moreover, pulmonary fibrosis can be caused by genetic factors and other diseases such as autoimmune diseases, systemic sclerosis, or Sjogren’s syndrome. Smoking, the environmental exposure (e.g., silica, metal, or wood dust), viral infections, gastroesophageal reflux, and certain drugs are risk factors for pulmonary fibrosis [110]. Silicosis, a pulmonary fibrosis disease caused by inhalation of silica dust particles, is characterized by irreversible nodule formation, aberrant fibroblast or myofibroblast proliferation, and excessive accumulation of ECM [111]. Bleomycin (BLEO) and lipopolysaccharide (LPS) induced lung injury are also common methods to build pulmonary fibrosis models [108,112].

Accumulating studies suggested that Tβ4 played a protective role in lung fibrosis. The van Gieson staining images showed that Tβ4 treatment reduced total collagen content in C57BL/6 mice lung tissues with BLEO-induced fibrosis, indicating that Tβ4 had antifibrotic properties [113]. Conte et al. found that treatment of BLEO-induced CD1 mice with Tβ4 alleviated lung fibrosis at early stage but had no effect at a late stage [114]. They also demonstrated that reduced pulmonary total collagen content in this model after Tβ4 administration was associated with reducing the number of IL17-producing cells in the blood and IL-17 expression in the lung tissues [115]. Tian et al. found that IHC images showed that Tβ4 was upregulated in both human fibrotic lung tissues and LPS-induced lung injury mice model. In addition, persistent expression of Tβ4 by administrating adeno-associated virus-Tβ4 intraperitoneally in LPS-induced mice model relieved lung inflammation and fibrosis. In cultured HPAEpiC and HLF-1 cells in vitro, Tβ4 notably inhibited the fibrogenic process such as EMT, mitophagy inhibition, and inflammasome activation [108]. 

POP reduces lung fibrosis through regulating Ac-SDKP synthesis. Li et al. suggested that treatment of S17092 decreased the concentration of Ac-SDKP through blocking Tβ4-POP-Ac-SDKP axis and restored pulmonary fibrosis in mutational mice expressing inactivated N-terminal catalytic sites of ACE with the induction of BLEO [116]. 

Ac-SDKP has preventive and therapeutic effects on lung fibrosis. Studies showed that pre-treatment and treatment of Ac-SDKP significantly decreased collagen deposition in lung tissues of silicosis rats, suggesting that Ac-SDKP could not only reduce the progression but also prevent the occurrence of pulmonary fibrosis [117].

According to research findings, Ac-SDKP inhibited TGF-β signaling transduction pathway, increased acetylated α-tubulin (α-Ac-Tub) expression, and interfered renin–angiotensin system (RAS) as crucial elements of its anti-fibrotic action.

Differentiation of fibroblasts into myofibroblasts and EMT are important in the development of fibrosis and this process can be activated by TGF-β1 [118,119]. Conte et al. reported that in CD-1 mice administrated with BLEO intratracheally, Ac-SDKP treatment ameliorated lung fibrosis and collagen deposition of lung tissues and this effect was related to lower expression of TGF-β, IL-17, and α-SMA [112]. In addition, it was demonstrated that pre-treatment and treatment of Ac-SDKP had the same protective effect in vivo rat silicotic lungs via inhibiting the expressions of TGF-β and its receptor serum response factor (SRF) which is needed for myofibroblast differentiation. In vitro, Ac-SDKP pre-treatment in TGF-β-induced rat primary pulmonary fibroblasts decreased myofibroblast differentiation and collagen deposition [18]. Another study reported that in primary human lung fibroblasts isolated from IPF, TGF-β-induced α-SMA expression, and collagen synthesis, which are key indicators of fibroblast differentiation into myofibroblasts, were markedly inhibited by Ac-SDKP, the hydrolysate of Tβ4 [114]. Deng et al. reported that pretreatment and treatment with Ac-SDKP both decreased collagen I and III deposition in lung samples of silicotic fibrosis rats. They further demonstrated that Ac-SDKP alleviated fibrosis through inhibiting the transition of epithelial cells to myofibroblasts mediated by activating the TGF-β1/ ROCK1 pathway in rat silicosis model and in cultured human embryo lung fibroblast MRC-5 and the human alveolar epithelial cell line A549 exposed to TGF-β1 [120]. Moreover, by interfering the TGF-β1-mediated the Rho-associated coiled-coil forming protein kinase (ROCK) signaling pathway, Ac-SDKP inhibited the differentiation of primary pulmonary fibroblasts from rats to myofibroblasts and collagen production [121]. Other studies indicated that treatment of Ac-SDKP inhibited myofibroblast differentiation and collagen formation both in silicotic rats in vivo and cultured A549 cells with TGF-β1 induction by reducing the levels of phosphorylated heat shock protein 27 (HSP27) and zinc finger family transcriptional repressor 1 (SNAI1), which are important regulators in EMT process [122,123].

Ac-SDKP also alleviates fibrosis by regulating other TGF-β signaling transduction pathways. Ramasamy et al. reported that Ac-SDKP inhibited the elevated expression of TGF-β and the phosphorylation of Smad3 in Ang II and ET-1-induced lung fibroblasts WI-38 cells [124]. Sun et al. found that chronic administration of Ac-SDKP prevented the collagen I and III synthesis in SiO_2_-induced silicosis rat model in vivo and TGF-β1-stimulated primary pulmonary fibroblasts in vitro and these effects are associated with inhibition of macrophage infiltration, TGF-β1 expression, and its downstream c-jun N-terminal kinase (JNK) phosphorylation and translocation to the nucleus [125]. Wei et al. reported that Ac-SDKP treatment prevented interstitial collagen accumulation and JNK signaling pathway activation which is mediated by TGF-β1 in a rat model of silicosis and cultured rat pulmonary fibroblasts [126]. Additionally, Ac-SDKP treatment inhibited TGF-β receptor-mediated p38 MAPK pathway to reduce fibrosis both in rats with silicosis and cultured pulmonary fibroblasts from neonatal rats [127]. Other studies showed that in a silicotic rat model, Ac-SDKP ameliorated pulmonary fibrosis by downregulating TGF-β1 and CTGF expression or inhibiting TGF-β1-induced Ras-Raf-ERK1/2 signaling pathway [128,129]. 

Wang et al. reported that Ac-SDKP administration played a therapeutic role in pulmonary fibrosis by downregulating histone deacetylase family member 6 (HDAC6) and reversed the reduced expression of α-tubulin acetyltransferase 1 (α-TAT1) in silicosis disease rat model and primary rat lung fibroblasts treated by Ang II to stabilize α-Ac-Tub expression level, resulting in inhibition of myofibroblast differentiation and collagen generation [130]. Exogeneous Ac-SDKP was also found to suppress myofibroblast differentiation and ECM production both in silicotic rat model and vitro pulmonary fibroblasts via inhibiting HDAC6 and increasing α-Ac-Tub expression [131]. Li et al. found that the collagen accumulation and α-SMA expression were suppressed by Ac-SDKP in rats exposed to silica and α-TAT1 expression was increased after Ac-SDKP treatment in this model. They further demonstrated that in vitro, Ac-SDKP inhibited lung fibrosis and induced cell apoptosis associated with upregulating α-TAT1 in the human type II alveolar epithelial A549 cell line and human embryo lung fibroblast MRC-5 cell line induced by TGF-β1 and SiO_2_ [132].

Disturbing the homeostasis of the RAS is a major component in the development of pulmonary fibrosis [133]. The Ang II produced by ACE is the primary factor driving RAS to exert effects. Ang II overproduction following chronic lung injury can activate fibroblasts leading to silicosis [18]. Moreover, Ang II can be converted to Ang-(1–7) by ACE2 and then binds to the Mas receptor and inhibits myofibroblast differentiation induced by TGF-β or Ang II to exert antifibrotic effects [134,135]. A study showed that Ac-SDKP suppressed ACE–Ang II–Ang II type 1 receptor (AT1) axis and activated ACE2-angiotensin-(1-7) [Ang-(1-7)]-Mas axis in silicotic C57BL/6 mice and Ang II-stimulated mouse lung type II epithelial cells MLE-12 to reduce EMT and alleviate lung fibrosis [111]. Gao et al. found that Ac-SDKP decreased pulmonary ECM deposition by increasing the level of ACE2-Ang-(1–7)-Mas axis in a silicotic rat model and Ang II-induced cultured lung fibroblasts [136]. Ac-SDKP could target RAS to attenuate fibrosis in silicotic rat lung tissues and cultured human embryonic lung cell line MRC-5 fibroblasts treated by Ang II and this effect was associated with inhibition of Hh signaling pathway [137]. Zhang et al. reported that decreased Ac-SDKP expression level and enhanced ACE, Ang II, and AT1 levels led to the formation and progression of silicotic fibrosis in rats exposed to silica. They also discovered that Ac-SDKP inhibited myofibroblast differentiation in rats with silicosis and in primary lung fibroblasts in vitro with the induction of Ang II [138]. Xu et al. reported treatment and pre-treatment of Ac-SDKP alleviated the elevation of AT1 in rats with silicotic fibrosis and inhibited RAS signaling [18].

Additionally, Ac-SDKP played the role of anti-pulmonary fibrosis via inhibiting alveolar epithelial cell apoptosis, inflammation, and glycolysis. A study indicated that through reducing Caspase-12 and PERK/eIF2/CHOP pathway activation triggered by endoplasmic reticulum (ER) stress, Ac-SDKP ameliorated apoptosis of vitro human A549 cells induced by SiO_2_ and vivo type II alveolar epithelial cells (AECs) in lung tissues of silicotic rats, resulting in attenuating silicotic fibrosis [139]. Jin et al. suggested that Ac-SDKP alleviated pulmonary fibrosis through decreasing macrophage activation and inflammatory reaction mediated by inhibiting the toll-like receptor 4 (TLR4) and receptor activator of nuclear factor kappa-B ligand (RANKL) signaling pathways in silicotic rats, cultured NR8383 alveolar macrophages, and the RAW 264.7 murine monocyte/macrophage cell line treated by silica [140]. Mao et al. demonstrated that in silicotic rats and silica-induced NR8383 alveolar macrophages, Ac-SDKP treatment reduced the enhanced expression of the important glycolysis enzymes HK2, PKM2, LDHA, and macrophage activation factors iNOS, TNF-α, Arg-1, IL-10, and MCP1, suggesting that Ac-SDKP can inhibit macrophage activation and glycolytic reprogramming to play the role of anti-inflammation and anti-fibrosis [141].

## 7. Conclusions

**Figure 2:**
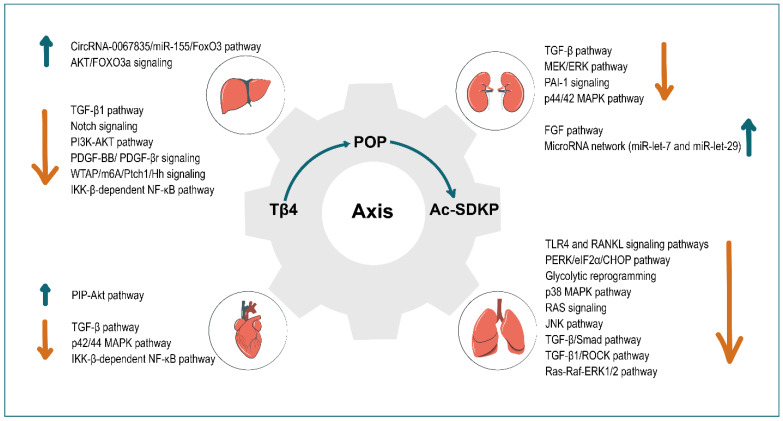
The signaling pathways regulated by Tβ4-POP-Ac-SDKP axis to inhibit liver, kidney, heart, and lung fibrosis. Up arrow represents upregulation, down arrow represents downregulation. Note: TGF-β, transforming growth factor-β; WTAP, Wilms’ tumour 1-associated protein; m6A, N6-methyladenosine; Hh, Hedgehog; NFκB, nuclear factor-κB; circRNA, circular RNA; miRNA microRNA; PDGF, platelet-derived growth factor; FGF, fibroblast growth factor; MAPK, mitogen-activated protein kinase; PAI-1, plasminogen activator inhibitor-1; PIP, PINCH-1-ILK-α-parvin; Smad, Suppressor of Mothers Against Decapentaplegic Miscellaneous; ROCK, Rho-associated coiled-coil forming protein kinase; JNK, c-jun N-terminal kinase; RAS, renin–angiotensin system; TLR4, toll-like receptor 4; RANKL, receptor activator of nuclear factor kappa-B ligand.

The Tβ4-POP-Ac-SDKP axis is an important protective mechanism against fibrosis in different organs in the early and late stages. The early stage refers to the fibrogenic stage when fibrosis is not severe and organ fibrosis has not yet formed. The late stage refers to the stage when organ fibrosis has established and is more severe. POP acts as an intermediate bridge on this axis. When this link of Tβ4 and Ac-SDKP is suppressed by POP inhibitor, Tβ4 will lose its anti-fibrotic effect in the late stage and promote fibrosis. It suggests that Tβ4 exerts an anti-fibrotic effect mainly in the late stage by promoting the accumulation of Ac-SDKP from the axis rather than Tβ4 itself [56]. However, in the BLEO-induced pulmonary fibrosis model, exogenous Tβ4 exerted a protective role in the early stage rather than the late stage [114]. Therefore, the effect of Tβ4 varies in different organs, and may be related to the activity of POP. Ac-SDKP plays a dominant role in anti-fibrosis in both early and late stages [57,92]. The absence or inhibition of any part of this axis, such as knockout of Tβ4, POP inhibitor administration, will aggravate fibrosis and this can be rescued by exogenous Ac-SDKP or ACE inhibitors to increase the concentration of Ac-SDKP [12,54,56,57]. Overall, pre-treatment and treatment of Ac-SDKP will improve fibrosis in both early and late stages, and Tβ4+Ac-SDKP may have a better anti-fibrotic effect in the late stage. Moreover, the Tβ4-POP-Ac-SDKP axis regulates the fibrogenesis via different cells such as HSCs, fibroblasts, or myofibroblasts which produce ECM and are involved in various pathways. The axis can mainly suppress the TGF-β/Smad signaling pathway and other TGF-β signaling transduction pathways such as the JNK signaling pathway and Ras-Raf-ERK1/2 signaling pathway. It downregulates the expression of TGF-β1, TGF-βRII, and TGF-β-induced expression of PAI-1, Smad2/3 phosphorylation, and promotes the PPARγ and translocation of Smad7. It also activates FGFR1 signaling by preventing the EMT or EndMT, promotes some microRNA expressions, and inhibits NF-κB pathway, Hh signaling, and Notch signaling, Notch2, and Notch3 to relieve the fibrosis. In addition, the axis also inhibits fibrosis by regulating the homeostasis of RAS and the physiological process of cells (Figure 2).

Treatment with exogeneous Tβ4 and Ac-SDKP has been demonstrated to have therapeutic benefits for fibrosis in various animal and cell models, with the aim of pushing new strategies into clinical trials. This review provides a comprehensive summary of the mechanisms underlying the protective effects of Tβ4-POP-Ac-SDKP axis on organ fibrosis and concludes an appropriate dosing regimen dependent on this axis for the early and late stages of fibrosis. In conclusion, it provides an indication for the application of Tβ4-POP-Ac-SDKP axis in appropriate dosing periods and regimens of organ fibrosis.

## Data Availability

Not applicable.
